# Portable Pulsed Coherent Lidar for Noncooperation Targets at the Few-Photon Level

**DOI:** 10.3390/s21072341

**Published:** 2021-03-27

**Authors:** Chengkai Pang, Qiongqiong Zhang, Zhaohui Li, Guang Wu

**Affiliations:** 1 State Key Laboratory of Precision Spectroscopy, East China Normal University, Shanghai 200062, China; 52180920012@stu.ecnu.edu.cn (C.P.); 52190920026@stu.ecnu.edu.cn (Q.Z.); zhhli@lps.ecnu.edu.cn (Z.L.); 2 Collaborative Innovation Center of Extreme Optics, Shanxi University, Taiyuan 030006, China

**Keywords:** coherent detection, laser ranging and imaging, decoherence

## Abstract

The decoherence in coherent lidar becomes serious with the increase in distance. A small laser spot can suppress the decoherence of the echo light from noncooperation targets. However, it is very difficult to keep a small light spot over a long distance. In this paper, a pulsed coherent lidar with high sensitivity at the few-photon level was demonstrated. A phase plate was used to modulate the wavefront of the laser to achieve 100 m focusing which reduced the decoherence effect. Based on coherent detection and time-of-flight (TOF) measurements, long-distance laser ranging and imaging on all days was realized. A signal classification and superposition method was used to extract the echo signal submerged in noise. The system was experimentally demonstrated by ranging different noncooperation targets within 105.0 m. The measurement rate was 10 k/s, and the measurement uncertainty was 1.48 cm. In addition, laser imaging was realized at ~50.0 m. The system was simple and portable as well as eye safe, and it may offer new application possibilities in automated vehicle lidar.

## 1. Introduction

Laser detection and ranging (lidar) has been widely used in topographic mapping, ocean detection, unmanned driving and other fields due to its advantages of high transmission efficiency, small size, and high resolution [1,2,3]. Recently, ranging and imaging lidar of tens of kilometers has been realized based on a single-photon detector, which is one of the most sensitive optical detection techniques [4,5,6,7,8,9]. Under strong background light, a single-photon detector will output a large number of noise counts or even saturation. On the other hand, coherent detection can effectively suppress the influence of background light by interfering with the received signal with a local oscillator, making it one of the most sensitive optical detection techniques [10,11]. It is widely used in Doppler lidar, coherent laser communication and other fields [12,13,14,15,16,17,18,19,20,21]. Pulsed coherent lidar has reduced the range ambiguity for long-distance ranges [22,23,24]. Coherent light becomes partially or completely incoherent, due to the random phase change of the wavefront, which is called decoherence. As the distance increases, the laser spot becomes larger and the decoherence becomes more serious. A small light spot can suppress the decoherence of the echo light from noncooperation targets. However, the laser spot of the lidar is usually larger than 20 mm in diameter while the distance is longer than 100.0 m [6,9]. It is very difficult to keep a small light spot over a long distance.

In this paper, a pulsed coherent lidar with high sensitivity at the few-photon level was proposed and experimentally demonstrated. Time-of-flight (TOF) measurement technology usually uses pulsed laser to irradiate the target, and then measures the flight time of the laser to get the distance of the target. Coherent detection can achieve high-sensitivity optical detection under strong background light. In the phase-shifted lidar system, the phase modulation period is usually milliseconds or even longer, which limits the data refresh rate. The refresh rate of TOF measurement is mainly limited by the pulse repetition rate [25,26,27], which is up to ~10^5^ per second in our system. Moreover, a phase plate was used to modulate the wavefront of the laser to achieve 100 m focusing so that the light spot was kept at approximately 10.0 mm in diameter, which reduced the decoherence effect. During the data processing, signal classification and superposition was used to extract the echo signal that was submerged in noise. The system was experimentally demonstrated by ranging different noncooperation targets within 105.0 m and imaging at ~50.0 m. The measurement uncertainty was 1.48 cm. The system was eye safe, portable and exhibited excellent anti-interference performance, which provides a new technology in automated vehicle lidar and site investigation.

## 2. Materials and Methods

### 2.1. Principle Analysis

**Figure 1:**
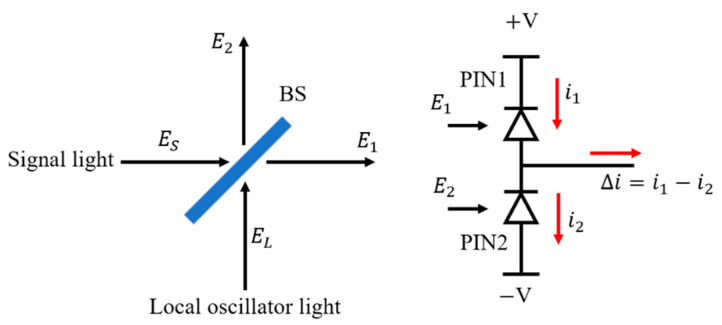
Balance detection.

In coherent detection, the signal and local beams satisfy the equations

(1) $$\left\{ \begin{array}{l} E_{S}=A_{S}\cdot \cos(\omega_{S}t+\varphi_{S})\\ E_{L}=A_{L}\cdot \cos(\omega_{L}t+\varphi_{L}) \end{array}\right.,$$

where *A_L_* and *A_S_* represent the amplitudes of the local oscillator light and signal light, respectively, and $\omega_{S}t+\phi_{S}$ and $\omega_{L}t+\phi_{L}$ are the phases of the two lasers at time *t*. The principle of balance detection is shown in Figure 1— photodetector1 (PIN1) and photodetector2 (PIN2) form a balanced detector (BD). After passing through the beam splitter (BS), the transmitted light of signal light and the reflected light of local oscillator light produce heterodyne interference on the photodetector1 (PIN1) and output photocurrent $i_{1}$, and the photodetector2 (PIN2) and output photocurrent $i_{2}$ in the same way. The signals of PIN1 and PIN2 are subtracted by a subtractor, and the output photocurrent of BD is Δi. Assuming that the BS has no optical loss, the transmissivity T and reflectivity R meet the requirements

(2) $$\left\{ \begin{array}{l} {\left|T\right|}^{2}+{\left|R\right|}^{2}=1\\ \left|\phi_{R}-\phi_{T}\right|=\pi /2 \end{array}\right.,$$

where $\phi_{R}$ and $\phi_{T}$ are the phase changes caused by reflection and transmission. Suppose the transmitted light is π/2 ahead of the reflected light after passing through a 50:50 BS, $E_{1}$ and $E_{2}$ can be expressed as:

(3) $$\left\{ \begin{array}{l} E_{1}=\frac{\sqrt{2}}{2}A_{S}\cdot \cos(\omega_{S}t+\varphi_{s}+\frac{\pi }{2})+\frac{\sqrt{2}}{2}A_{L}\cdot \cos(\omega_{L}t+\varphi_{L})\\ E_{2}=\frac{\sqrt{2}}{2}A_{S}\cdot \cos(\omega_{S}t+\varphi_{s})+\frac{\sqrt{2}}{2}A_{L}\cdot \cos(\omega_{L}t+\varphi_{L}+\frac{\pi }{2}) \end{array}\right..$$

The instantaneous photocurrent of BD satisfies the following formula

(4) $$\Delta i=i_{1}-i_{2}=\alpha {\left|E_{1}\right|}^{2}-\alpha {\left|E_{2}\right|}^{2}=\alpha A_{S}A_{L}\sin[(\omega_{L}-\omega_{S})t+(\varphi_{L}-\varphi_{S})]$$

where α is the responsivity of photodetector. It can be seen from Formula (4) that the photocurrent of BD contains the amplitude, frequency and phase information of the signal light. It is a special form of coherent detection when $\omega_{S}=\omega_{L}$, which is called homodyne detection.

In the direct detection, the signal light is directly incident on a photodetector, and the average photocurrent output by the detector is

(5) $$\bar{i}=\alpha \bar{{\left|E_{S}\right|}^{2}}=\frac{1}{2}\alpha A_{S}^{2}.$$

The photocurrent of direct detection is only related to the intensity of the signal light, and the detection is unable to be realized when the signal light is very weak. The photocurrent of balance detection is related to the intensity of both the signal light and the local oscillator light. A better output can be obtained by increasing the optical power of local oscillator light properly when the signal light is weak. Therefore, the coherent balance detection method has higher sensitivity compared with direct detection. Through the TOF measurement method, the generator produced a reference pulse signal, and the distance *L* can be calculated by L=ct/2 according to the relative time *t* between the echo signal and the reference pulse signal.

### 2.2. Signal Classification and Superposition

With increasing measurement distance, the energy of the echo signal decreases greatly. The method of multiple pulse superposition is generally used in photon-counting lidar to enhance the echo signal. In the case of $\omega_{S}=\omega_{L}$, the signal voltage $V_{S}$ on the load resistance $R_{L}$ after multiple pulse superposition in coherent detection can be obtained by Equation (4), which is

(6) $$V_{S}=\alpha R_{L}A_{S}A_{L}\int \sin[\Delta \varphi (t)]dt.$$

The local oscillator light is in a single-mode fiber in the whole process and the phase of the local oscillator light remains unchanged after the same displacement in a short period of time, thus $\varphi_{L}$ can be regarded as a constant. The phase of the signal light at the receiving end is changed because of the temperature, humidity, air flow and other factors in the space when the signal light passes through a long space distance. The phase difference Δφ(t) between signal light and local oscillator light can be expressed as

(7) $$\Delta \varphi (t)=\varphi_{L}-\varphi_{S}(t),$$

where $\varphi_{s}\left(t\right)$ is a random variable, thus sin[Δφ(t)]∈[−1,1], $\bar{V_{S}}\to 0$, no effective signal can be obtained after multiple pulse superposition in coherent detection.

In this paper, the method of signal classification and superposition is used to extract the echo signal submerged in noise, which increases the signal-to-noise ratio (SNR) and improves the sensitivity of the system. The voltage after superposition can be expressed as

(8) $$\left\{ \begin{array}{l} V_{+}=\alpha R_{L}A_{S}A_{L}\int \sin[\Delta \varphi (t)]dt,\Delta \varphi \in [0,\pi ]\\ V_{-}=\alpha R_{L}A_{S}A_{L}\int \sin[\Delta \varphi (t)]dt,\Delta \varphi \in (\pi ,2\pi )\\ V_{S}^{\prime }=V_{+}-V_{-} \end{array}\right..$$

Here, $V_{+}$ is the voltage value of positive signal superposition, $V_{-}$ is the voltage value of negative signal superposition, and $V_{S}{}^{\prime }$ represents the final signal voltage value. The average value of signal voltage $\bar{V_{S}{}^{\prime }}\to \alpha R_{L}A_{S}A_{L}/2$ when the sample size is large enough.

The noise in coherent detection lidar mainly comes from the shot noise $i_{1}$, the thermal noise $i_{2}$ introduced by photodetector and noise $i_{3}$ introduced by the amplifier. In data processing, *N* signals are classified and superimposed so that the actual noise is $\sqrt{N}$ of the original noise. For the whole system, the introduced noise voltage $V_{n}$ satisfies

(9) $$V_{n}=\frac{R_{L}\cdot i_{n}}{\sqrt{N}}=\frac{R_{L}\cdot \sqrt{i_{1}^{2}+i_{2}^{2}+i_{3}^{2}}}{\sqrt{N}}.$$

The signal-to-noise ratio (SNR) can be expressed as

(10) $$SNR=\frac{S}{N}=\frac{\bar{V_{S}^{\prime }}}{V_{n}}=\frac{\sqrt{N}\alpha A_{S}A_{L}}{2\sqrt{i_{1}^{2}+i_{2}^{2}+i_{3}^{2}}}.$$

### 2.3. Experimental Setup

**Figure 2:**
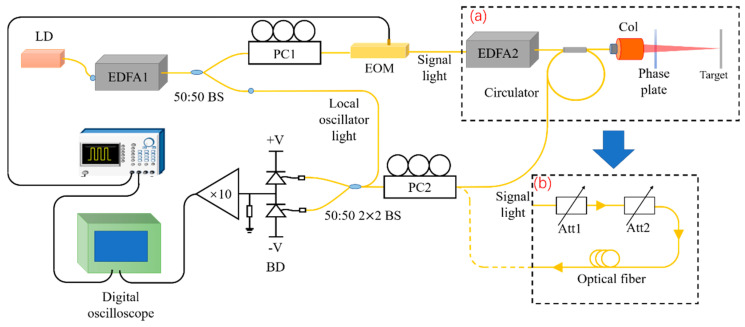
Experimental setup with (**a**) distance measurement system and (**b**) performance test system. LD: Laser diode; EDFA: erbium-doped fiber amplifier; BS: beam splitter; PC: polarization controller; EOM: electrooptic modulator; Col: collimator; BD: balance detector; Att: adjustable attenuator.

The experimental setup is shown in Figure 2, where part (a) is the distance measurement system. The seed light was produced by a 1550 nm laser diode (LD), and the power was 1.0 mW. The seed power was amplified by the first erbium-doped fiber amplifier (EDFA1). The amplified seed light was evenly divided into two paths by the 50:50 beam splitter (BS). One path was used as the local oscillator light and entered a 50:50 2 × 2 coupler, the other was used as the signal light. The generator generated a modulation signal and modulated the signal light into pulse light by using a polarization controller (PC1) and electrooptic modulator (EOM). After being amplified by EDFA2, the modulated signal light entered the circulator, and the output terminal was connected to the large aperture collimator (Thorlabs, C80APC-C). A phase plate was used to modulate the wavefront of the laser to achieve 100 m focusing so that the light spot was kept at approximately 10 mm in diameter, which reduced the decoherence effect. The diffuse signal light entered the 50:50 2 × 2 coupler through a circulator and the polarization controller (PC2) and interfered with the local oscillator light. The interference signal was received by a balanced detector (BD), and the detection signal was displayed and collected by a digital oscilloscope (DS09054H, Agilent, CA, USA) after being amplified by an operational amplifier. The balance detector consists of two InGaAs PIN photodiodes, whose responsivity is 0.9 A/W @1550 nm with the bandwidth of 2.5 GHz. Part (b) of Figure 2 shows the performance test system—the signal light entered the PC2 after being attenuated by two attenuators (Att1 and Att2) and then entered a 50:50 2 × 2 coupler. The power of the signal light entering Att1, measured by an optical power meter, is $P_{S}$; the attenuation coefficient of Att1 and Att2 is $A_{1}$ and $A_{2}$, respectively; the photon numbers of signal light received by BD can be obtained by the following formula

(11) $$\frac{P_{S}}{A_{1}\cdot A_{2}}=n_{ph}\frac{hc}{\lambda },$$

where *n_ph_* is the number of photons.

## 3. Results and Discussion

### 3.1. Method Verification

**Figure 3:**
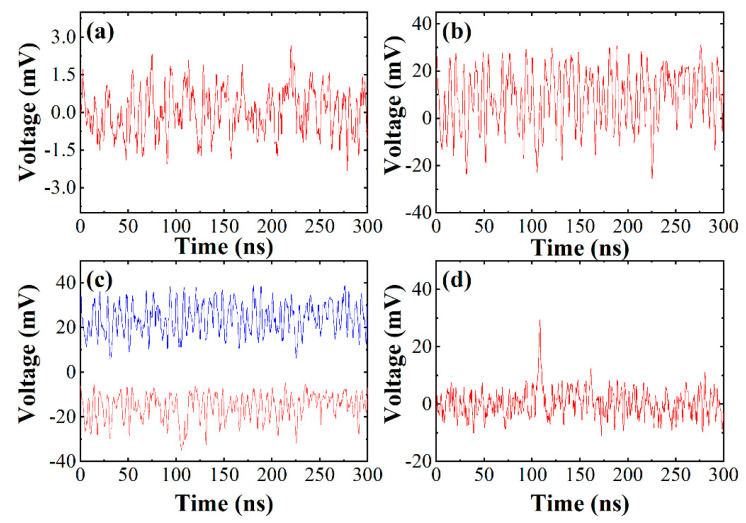
(**a**) Signal collected in a single period, (**b**) signal superposed in 50 periods, (**c**) signals after superposition of positive and negative separately, (**d**) signal after processing.

In this section, the method of signal classification and superposition was verified by an experiment for which the setup is shown in part (b) of Figure 2. In the verification process, the local oscillator light was 50.0 mW and the signal light was 5.5 photons per pulse on average. The pulse width of the signal light was 3 ns. Figure 3a shows the signal collected in a single period, and Figure 3b shows the signal superposed in 50 periods. Both pulse signals are completely submerged in the background signal, and the effective signals cannot be extracted. Figure 3c shows signals after superposition of positive and negative signals separately—the blue curve represents a positive signal, and the red curve represents a negative signal. Figure 3d shows the final signal after processing. Through the signal classification and superposition, the echo signal was significantly enhanced, which was helpful to improve the system performance.

**Figure 4:**
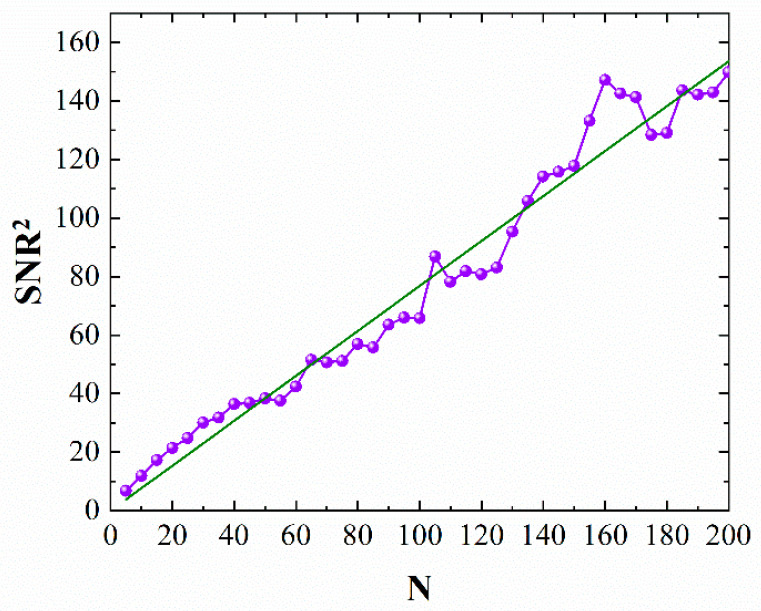
The experimental results of signal-to-noise ratio (SNR)^2^ under different periods by using the method of signal classification and superposition.

The SNR performance was tested under the condition of 15.0 mW local oscillator light and 300 photons per pulse signal light. The pulse width of the signal light was 3 ns. The SNR was tested from 5 periods to 200 periods every 5 periods, the setup is shown in part (b) of Figure 2, and the results are shown in Figure 4. The purple curve shows the experimental results, which can be fitted into a green straight line shown in Figure 4. The experimental results show that $SNR^{2}$ is directly proportional to the number of periods, which is consistent with Equation (10).

### 3.2. Performance Test

**Figure 5:**
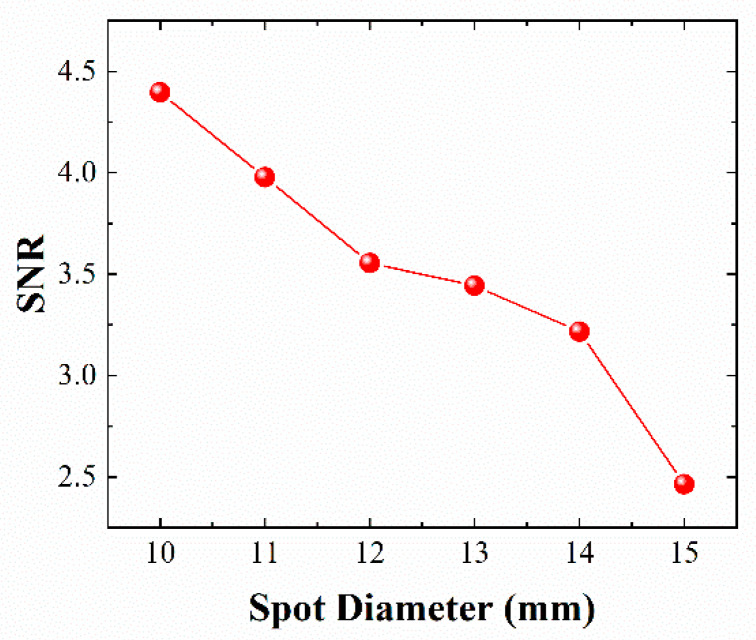
SNRs with different spot sizes under the same measurement conditions.

In a coherent lidar system, the light spot becomes larger with increasing distance, which leads to serious decoherence. SNRs with different spot sizes were tested under the condition of 8.1 mW local oscillator light and 0.7 mW signal light, and the setup is shown as part (a) of Figure 2. The pulse width of signal light was 6 ns, the measurement distance was 5.0 m, and the results are shown in Figure 5. The SNR decreases with increasing spot diameter. Therefore, a small spot size can improve the SNR of coherent lidar and achieve high-sensitivity detection. The phase plate modulates the wavefront of the incident light through the micro-relief structure on the surface of the substrate, which redistributes the complex amplitude of the light wave field in space and obtains the expected output light field. In the experiment, a phase plate was used to modulate the wavefront of the light spot with a diameter of approximately 14.5 mm to achieve 100 m focusing. The spot diameter was kept within 10.0 mm in the range of 50.0–120.0 m, and 100 m level ranging and imaging of pulsed coherent lidar with high sensitivity was realized.

**Figure 6:**
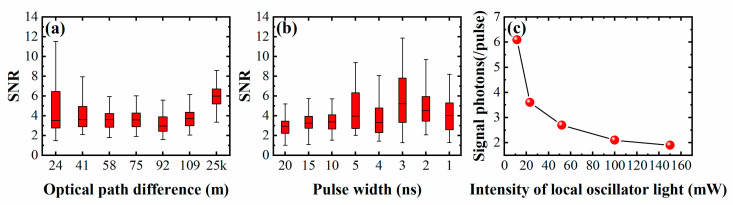
(**a**) SNR at different optical path difference; (**b**) SNR at different pulse width; (**c**) the test of signal photon-number detection limit under different intensities of local oscillator light.

The performance of this system was tested by a quantitative control method with the signal superposed in 50 periods; the setup is shown as part (b) of Figure 2. The laser coherence was tested under the condition of 15.0 mW local oscillator light and 300 photons per pulse signal light. The pulse width was 3 ns, and the optical path difference of the system could be changed by changing the length of the optical fiber in Figure 2. The fiber length was increased from 24.0 m to 25 km, and the corresponding SNR value is shown in Figure 6a. The system still has good SNR when the optical path difference is increased to 25 km, which can effectively distinguish the signal pulse. Therefore, the laser used in this experiment had excellent interference, which completely met the requirement of laser ranging based on coherent detection within 105.0 m. The SNR remains basically unchanged, and the abnormal change at 25 km may come from the random fluctuation of phase. Then, the influence of different pulse widths of signal light on SNR was tested under the condition of 15.0 mW local oscillator light and 300 photons per pulse signal light. The optical path difference was 24.0 m and the energy of each pulse remained unchanged in the testing process. The results are shown in Figure 6b, the SNR shows an increasing tendency with the decrease in the pulse width. Therefore, a smaller pulse width was beneficial for improving the measurement results during application. Finally, the signal photon-number detection limit was tested under different intensities of local oscillator light. The pulse width of the signal light was 3 ns, the optical path difference was 24.0 m, and the results are shown in Figure 6c. The slope of the curve decreases with the increase in the local oscillator light power, thus the decreasing trend of the photon-number detection limit gradually becomes indistinct.

### 3.3. Ranging and Imaging

**Figure 7:**
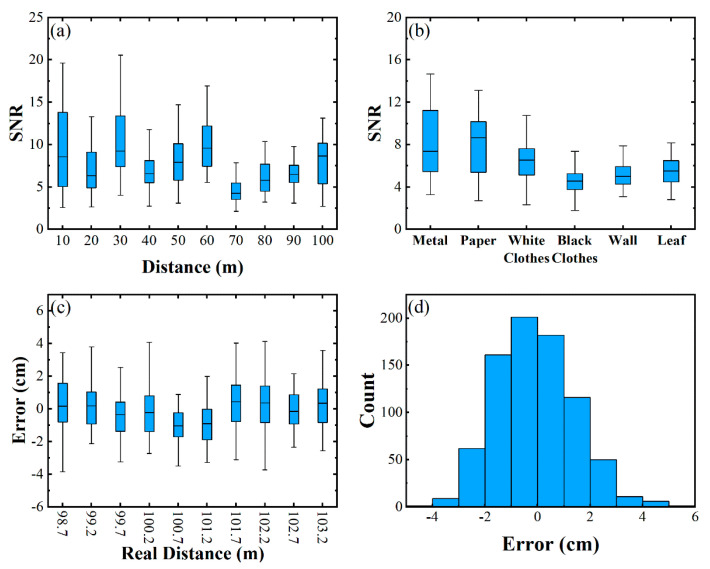
(**a**) SNR of paper at different distances, (**b**) SNR of different targets at 103.2 m, (**c**) uncer-tainty test near 100.0 m, (**d**) error distribution between measured value and actual value.

The experiment was carried out within 105.0 m, and the setup is shown in part (a) of Figure 2. In the ranging experiment, the local oscillator light power was 24.6 mW. The repetition rate of the signal light was 500 kHz, the pulse width was 6 ns and the power was ~1.6 mW. The SNR of paper at different distances within 105.0 m are shown in Figure 7a. With the increase in distance, the SNR of the target could be maintained in a good state. On the street, buildings, billboards, railings, vegetation and pedestrians are common targets; thus, the SNR of a wall, a metal plate, a piece of paper, a leaf, a white T-shirt (Adidas FI0882, cotton) and a black T-shirt (Adidas GJ6565, cotton) were tested at 103.2 m.

The results are shown in Figure 7b, and the target objects of different materials indicate good SNR. Therefore, this pulsed coherent lidar has the application potential of vehicle lidar. Finally, the uncertainty of the system was tested in the range of 98.7–103.2 m every 0.5 m, and each position was measured 80 times. The test results are shown in Figure 7c, and the error distribution between the measured distance and the actual distance is shown in Figure 7d, the standard deviation was 1.48 cm. The accuracy was mainly affected by the pulse width of echo signal and the SNR. In the range of 100.0 m, the echo pulse broadening could be ignored, and the SNR change was very small. Therefore, the measurement uncertainty was basically kept at 1.48 cm.

In addition, the experimental setup was adjusted by installing the collimator and the phase plate on an electric motor to achieve imaging. The pulsed coherent lidar was placed at the origin of the polar coordinate system since the laser was roated in horizontal and vertical directions. To obtain the image in the Cartesian coordinate system, the image was corrected by

(12) $$\left\{ \begin{array}{l} X=L\sin\theta \cos\phi \\ Y=L\sin\theta \sin\phi \\ Z=L\cos\theta \end{array}\right.,$$

where *L* is the distance between target and lidar calculated by TOF measurement, *θ* and *ϕ* are the scanning angle of the laser in the horizontal and vertical directions, respectively, corresponding to the position in the polar coordinate system, and *X*, *Y*, *Z* are the positions in the Cartesian coordinate system.

**Figure 8:**
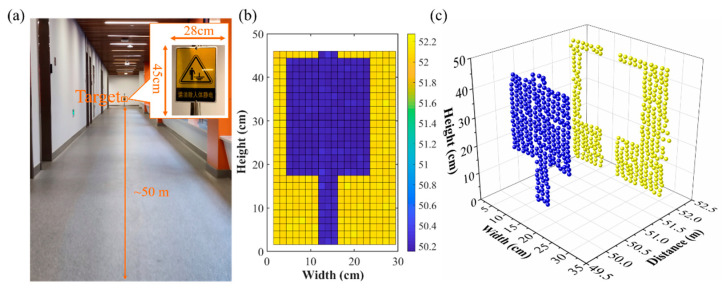
(**a**) The photograph of scanning target, (**b**) the front view of imaging picture, (**c**) the point cloud of imaging picture.

In this paper, a notice board was scanned at approximately 50.0 m in a corridor with good light conditions. The imaging picture is shown in Figure 8. The single steps in the X and Y directions were 0.29 mrad and 0.32 mrad, respectively. Figure 8a shows a photograph of the scanning target. The scanning area was 28 cm × 45 cm, and there was a 1.9 m distance difference between the notice board and the wall. Figure 8b shows the front view of the imaging picture with pixels of 20 × 29. The blue part is the notice board, and the yellow part is the wall. Figure 8c shows the point cloud of imaging picture. The uncertainty of the horizontal and vertical direction of the motor was 0.012 mrad and 0.014 mrad, respectively. Due to the coherence between the local oscillator light and the signal light, there was no noise point or even scanning during the day time. The pulsed coherent lidar could realize whole-time ranging and imaging. According to the standard of international electrotechnical commission (IEC 60825), the laser of this system was classified as class 1.

## 4. Conclusions

In this paper, a pulsed coherent lidar with high sensitivity was proposed and experimentally demonstrated. Based on coherent detection and TOF measurement, long-distance laser ranging and imaging on all days was realized. A signal classification and superposition method was used to extract the echo signal submerged in noise and a phase plate was used to modulate the light source to achieve 100 m focusing. The system was experimentally demonstrated by ranging different material targets within 105.0 m. In addition, laser imaging based on coherent detection was realized at ~50.0 m. The system was simple and portable and exhibited excellent anti-interference performance with eye safe, which opens new application possibilities in automated vehicle lidar and site investigation. In the further work, the narrow linewidth laser with high power and low noise can be used to improve the SNR of the system, and the embedded system can be developed to realize the real-time data processing, so that the pulsed coherent lidar can meet the requirements of autonomous vehicle application.

## Data Availability

Not applicable.
